# Occupational disparities in tumor grade and cytosolic HMGB1 expression in renal cell cancer

**DOI:** 10.1002/1348-9585.12340

**Published:** 2022-06-16

**Authors:** Masayoshi Zaitsu, Takumi Takeuchi, Masaaki Zaitsu, Akiko Tonooka, Toshimasa Uekusa, Yudai Miyake, Yasuki Kobayashi, Gen Kobashi, Ichiro Kawachi

**Affiliations:** ^1^ Department of Public Health Dokkyo Medical University School of Medicine Mibu, Tochigi Japan; ^2^ Department of Public Health Graduate School of Medicine The University of Tokyo Tokyo Japan; ^3^ Department of Urology Kanto Rosai Hospital Kawasaki Kanagawa Japan; ^4^ Center for Research of the Aging Workforce University of Occupational and Environmental Health Kitakyushu Fukuoka Japan; ^5^ Department of Gastroenterological Surgery Ⅰ Hokkaido University Graduate School of Medicine Sapporo Hokkaido Japan; ^6^ Division of Pathology Cancer Institute Japanese Foundation for Cancer Research Tokyo Japan; ^7^ Department of Pathology Kanto Rosai Hospital Kawasaki Kanagawa Japan; ^8^ Department of Social and Behavioral Sciences Harvard T.H. Chan School of Public Health Boston Massachusetts USA

**Keywords:** HMGB1, occupational disparity, renal cell cancer, tumor grade

## Abstract

**Objectives:**

We sought to examine occupational disparities in tumor grade and cytosolic expression of high‐mobility group box‐1 (HMGB1) among renal cell cancer (RCC) patients.

**Methods:**

This retrospective study included 318 RCC patients with complete information on occupation and pathology in Kanagawa Cancer Registry (KCR). Longest‐held occupations were grouped into manual workers (e.g., manufacturing, construction) versus “others.” Odds ratios (OR) and 95% confidence intervals (CI) for high‐grade histology were estimated by logistic regression, adjusted for age and sex. We also examined a sub‐sample of 74 low‐grade RCC inpatients to estimate the OR for positive cytosolic HMGB1 expression in manual workers, adjusting for age, sex, and other available covariates.

**Results:**

High‐grade tumors were more prevalent in manual workers compared to other occupations: 23.0% (14/61) versus 10.9% (28/257, *p* = .01) with an adjusted OR of 2.28 (95% CI, 1.11–4.69). In the sub‐sample of low‐grade RCCs, positive cytosolic HMGB1 expression was more prevalent in manual workers compared to other occupations: 71.4% (10/14) versus 38.3% (23/60, *p* = .03) with a sex‐ and age‐adjusted OR of 3.76 (95% CI, 1.03–13.7).

**Conclusions:**

Manual workers are associated with increased risks of high‐grade renal cell tumors and cytosolic HMGB1 expression.

## 1 INTRODUCTION

Renal cell cancer (RCC) is the sixth and tenth most common cancer worldwide in men and women, respectively.^1^ In Japan, the incidence rate for RCC is roughly one‐third compared with that in Western countries, but the incidence has been rising.^1^, ^2^ As in other malignant tumors, histologically high‐grade RCCs are associated with worse prognosis.^3^

The contribution of lifestyle metabolic risk factors (e.g., hypertension and diabetes) to RCC tumor grade has been debated.^4^, ^5^ There are known socioeconomic disparities in metabolic risk factors. Therefore, it would be possible that those in lower socioeconomic groups (e.g., manual workers) are linked to higher prevalence of metabolic risks, which may in turn result in higher RCC tumor grade in those socioeconomic strata. Within such lower socioeconomic groups, some specific occupations are also associated with cancer risk. For example, previous studies suggest that occupations in certain manufacturing industries (e.g., metal workers, printers, and oil processors) are associated with higher *incidence* of RCC.^2^, ^6^, ^7^, ^8^, ^9^ Workers in transportation are suggested to at risk of cancer.^10^, ^11^, ^12^ However, evidence remains sparse on socioeconomic disparities in RCC tumor grades.

Additionally, high‐mobility group box‐1 (HMGB1) is known to be a typical damage‐associated molecular pattern molecule (DAMP) triggering an immune response in combination with Toll‐like receptor (TLR) and the receptor for advanced glycation end products (RAGE).^13^ In the case of RCC, HMGB1 is thought to predict high‐grade tumors. The distribution of positive cytosolic HMGB1 expression was higher in patients with Grade 2 or greater RCC compared with those with Grade 1 RCC in a previous study (17/27 [63%] vs. 2/12 [17%], *p* = .01).^14^ However, little is also known about occupational differences in positive cytosolic HMGB1 expression among RCC patients. In this context, we hypothesized that even among low‐grade RCC patients, certain occupations would be associated with the positive cytosolic HMGB1 expression, a biological marker for high‐grade histological potential.^14^

In this retrospective study of RCC patients, using a population‐based cancer registry and inpatient immunohistochemistry data in Japan, we sought to examine whether there are occupational disparities in tumor grade and cytosolic HMGB1 expression.

## 2 MATERIALS AND METHODS

### 2.1 Study design, setting, and participants

In this cross‐sectional study, we analyzed a population‐based dataset (1974–2016) of the Kanagawa Cancer Registry (KCR). We also analyzed inpatient data (2008–2019) from Kanto Rosai Hospital, a designated hospital participating in the KCR. The patients registered in KCR and those in Kanto Rosai Hospital are both drawn from one metropolitan prefecture of Kanagawa Prefecture that covers ~7% of the Japanese population. Details for KCR, accredited by the Surveillance, Epidemiology, and End Results (SEER), and Kanto Rosai Hospital have been previously described.^14^, ^15^, ^16^, ^17^, ^18^ Briefly, both datasets included basic information (sex, age, date of diagnosis) and pathological information. The KCR collected information on occupation if available during the study period (~10% of the registered cases). Although the data of KCR spanned several decades, previous diagnosis and pathological codes are updated to the latest version to be consistent with changes in coding practice.^15^, ^16^, ^17^ For inpatient data on RCC from Kanto Rosai Hospital, occupational background was gathered from medical charts, and immunohistochemistry data of the cytosolic expression of HMGB1 were included to the dataset. The study was carried out using an opt out approach in accordance with the guidelines outlined in the Helsinki Declaration of 1964. De‐identified datasets were used in the present study, and the local ethics committees approved the study (Protocol Number 2020–004).

**FIGURE 1:**
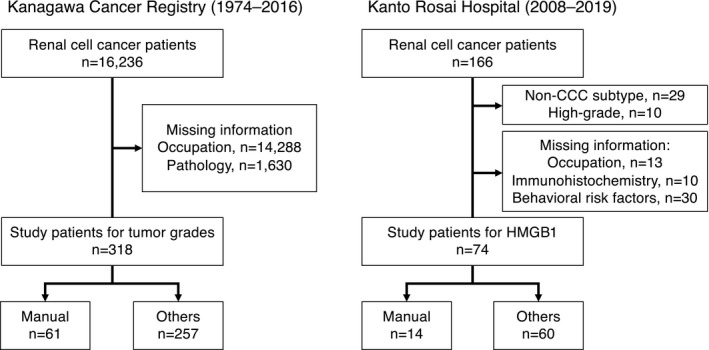
Flowcharts showing the selection of renal cell cancer patients in the Kanagawa Cancer Registry and Kanto Rosai Hospital. Abbreviation: HMGB1, high‐mobility group box‐1

For the main analysis of the association between occupation and histological grade, we analyzed RCC patients registered in the KCR (C64 in International Classification of Diseases, 10th revision). Of 16 236 patients, we included 318 patients who had complete information on pathological grade and occupation (Figure 1). For the immunohistochemistry data analysis, we analyzed inpatients with RCC in Kanto Rosai Hospital. Of 166 inpatients, we included 74 inpatients with histological subtype of low‐grade clear cell carcinoma (CCC), who had complete information on occupation, immunohistochemistry, and behavioral risk factors (such as smoking, drinking, and comorbidities; Figure 1). Due to a large fraction for missing data, imputation was not possible (Figure 1).

### 2.2 Definitions of primary and secondary endpoints

Our primary endpoint was RCC tumor grade. Among RCC patients registered in KCR having pathological diagnoses with the World Health Organization (WHO) pathological grading system,^16^, ^17^ we identified 42 patients with high‐grade tumors, having WHO pathological grade of 3 or 4. The remaining 276 were classified as low‐grade tumors, having WHO pathological grade of 1 or 2.

For the immunohistochemistry data analysis in the subsample of 74 low‐grade inpatients treated at Kanto Rosai Hospital (defined by WHO grade of 1 or 2), we identified 33 cases with positive expression of cytosolic HMGB1 in RCC specimens and 41 with negative expression of cytosolic HMGB1.

### 2.3 Occupational backgrounds

Using the longest‐held occupation recorded in KCR and Kanto Rosai Hospital based on the Japan Standard Occupational Classification, we classified each RCC patient into two groups: manual workers or “other” occupational group (i.e., non‐manual workers).^19^, ^20^ Manual workers included those involved in occupational classes of manufacturing, construction, mining, and transportation. “Other” occupational group included professional, managerial, clerical, sales, and service workers. According to a potential lower cancer risk in previous studies,^21^, ^22^ those who were not actively engaged in paid employment such as homemakers, students, unemployed, and class unknown workers were also grouped in the other occupational group. Industrial clusters were not specified in this study.

In sensitivity analysis, we used different patient grouping, considering possible misclassification of exposure to lifestyle related factors and carcinogen by our relatively broad classification for occupation.^2^, ^6^, ^7^, ^8^, ^9^ We excluded those involved in transportation from the manual worker group because they were less likely to expose to industrial agents. We also excluded those involved in service and sales from the other occupational group because they were also higher risk of lifestyle‐related factors compared to professional and managerial workers.^19^, ^23^

### 2.4 Immunohistochemistry data

**FIGURE 2:**
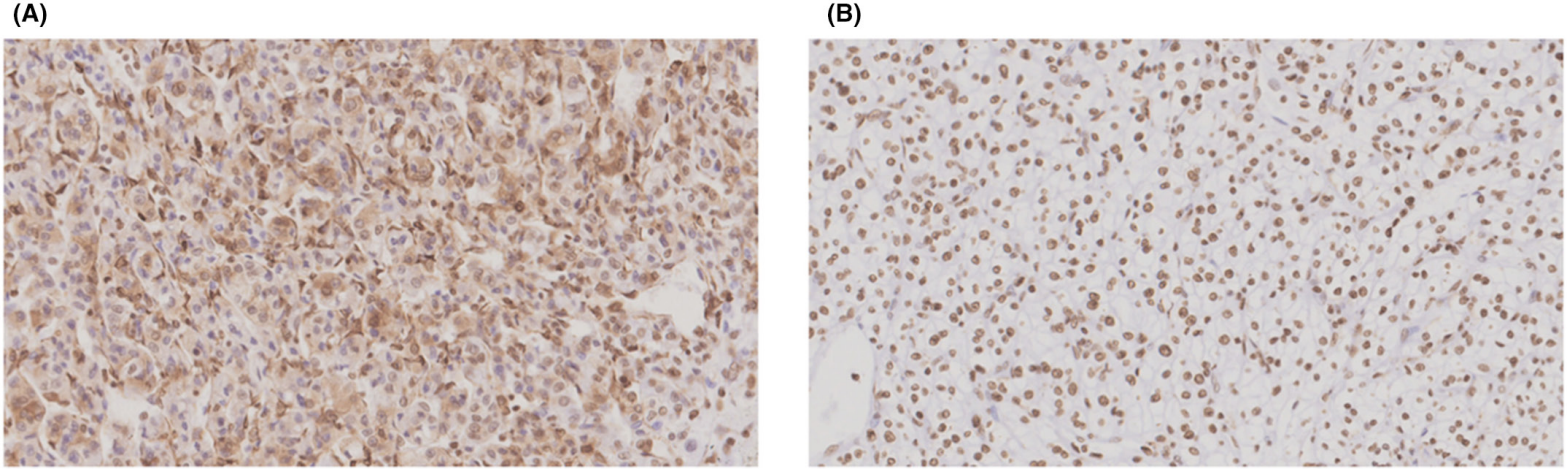
Immunohistochemistry staining high‐mobility group box‐1 among renal cell cancer patients. Positive cytosolic expressions (A) and negative expressions (B) in tumor cells

Following the methodology used in previous studies, 74 paraffin‐embedded sections of preserved RCC specimens, excised by radical or partial nephrectomy, were immunohistochemically stained in accordance with the manufacturers’ instructions.^14^ For primary anti‐HMGB1 antibodies, ab80246 (Abcam) was used for 12 specimens during 2008 to 2012. Additionally, we obtained immunohistochemical data from 62 specimens during 2012 to 2019, separately from the prior 12 specimens. In this additional process, ab79823 (Abcam) were used for the staining process at a professional immunohistochemistry testing company (Morphotechnology Co., Ltd, Hokkaido, Japan). An independent pathologist (AT or TU) confirmed the positive expression of cytosolic HMGB1, referring a positive control, in a double‐blinded manner if they judged that the cytosolic HMGB1 expressions were positive for more than 30% tumor cells on sections (Figure 2).^14^ We validated the judgment of two independent pathologists by the results of complete matching of diagnoses on five different specimens. Secondary antibodies were not used based on previous studies.^14^

### 2.5 Other variables

Age and sex were included as basic confounding variables. For potential mediating variables that may explain the association between occupation and tumor grade, we included histological subtype (CCC or non‐CCC [e.g., papillary, chromophobe, and sarcomatoid differentiation]) in the analysis for KCR.^14^, ^17^ For potential mediating variables that may explain the association between occupation and high‐grade potentials expressed by cytosolic HMGB1 in the analysis among low‐grade inpatients, we included smoking (pack‐years), drinking (drink‐years), hypertension (yes/no), diabetes (yes/no), and body mass index as occupation‐related behavioral factors.^1^, ^2^, ^4^, ^24^ Furthermore, we also included C‐reactive protein (CRP) as well as neutrophil‐to‐lymphocyte ratio (NLR) in blood test before nephrectomy as a systemic chronic inflammatory indicator that has been reported to play a critical role in pathogenesis and progression of cancer.^25^, ^26^, ^27^

### 2.6 Statistical analysis

The background characteristics were compared by a *t*‐test or chi‐squared test.

For KCR data, we estimated the odds ratio (OR) and 95% confidence interval (CI) of high‐grade tumors for manual workers using logistic regression, adjusted for sex and age (main analytic model). The other occupational group served as the reference group in all analyses. In addition, we adjusted for non‐CCC subtype as mediators.^28^

For immunohistochemistry data from the 74 inpatients, we estimated the OR of the positive cytosolic HMGB1 expression for manual workers, adjusted for age and sex (main analytic model). In addition, as a supplemental data analysis, we further adjusted for behavioral and chronic inflammatory covariates as mediators.^28^

In sensitivity analyses, we performed analyses just restricted to men. Due to limited sample size, we could not perform analyses in women only. Additionally, we used different patient grouping (excluding those involved in transportation, service, and sales), considering possible misclassification of exposure regarding lifestyle related factors and carcinogen by our relatively broad classification for occupation. In stratified analyses of immunohistochemistry data by primary antibodies, we restricted analyses to the 62 specimens collected during 2012 to 2019 (ab79823) because of potential differences in staining process of primary antibodies.

Alpha was set at 0.05, and all *p*‐values were two‐sided. Data were analyzed using STATA/MP13.1 (StataCorp LP, College Station, TX, USA).

## 3 RESULTS

### 3.1 Occupational differences in high‐grade tumors among RCC patients in KCR

**FIGURE 3:**
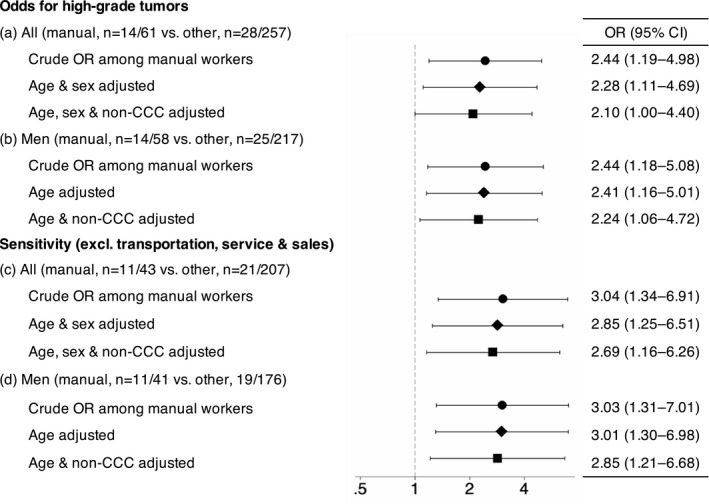
Odds ratios of renal cell cancer patients of manual workers for high‐grade tumors estimated by logistic regression. Abbreviations: CCC, clear cell carcinoma; CI, confidence interval; OR, odds ratio

Among RCC patients in KCR, high‐grade tumors were more prevalent in manual workers compared with others (Table 1): 23.0% versus 10.9% (*p* = .01). The age and sex‐adjusted odds for high‐grade tumors was elevated among manual workers: the OR was 2.28 (95% CI, 1.11–4.69; Figure 3). An increased OR was observed among manual workers after additional adjustment for histological subtype (Figure 3). The numbers of high‐grade tumors in specific blue‐collar occupations are shown in Table 2, showing higher percentage of high‐grade tumors among RCC patients of construction workers.

**TABLE 1:** Characteristics of renal cell carcinoma patients in Kanagawa Cancer Registry and a designated hospital who had complete information on occupation and histology

Characteristics	Mean ± SD or number (%)		*p*‐value
	Manual^a^	Others	
RCC patients in Kanagawa Cancer Registry, *n* = 318			
Total number	*n* = 61	*n* = 257	
High‐grade histology	14 (23.0)	28 (10.9)	0.01
Age	59 ± 10	58 ± 11	0.34
Female	3 (4.9)	40 (15.6)	0.03
Non‐clear cell carcinoma subtype	8 (13.1)	18 (7.0)	0.12
Patients with low‐grade clear cell carcinoma in Kanto Rosai Hospital, *n* = 74			
Total number	*n* = 14	*n* = 60	
Positive cytosolic HMGB1 expression	10 (71.4)	23 (38.3)	0.03
Age, yrs	66 ± 12	61 ± 12	0.24
Female	1 (7.1)	16 (26.7)	0.12
Smoking, pack‐years	12.6 ± 16.0	15.6 ± 18.4	0.57
Drinking, cup‐years	49.8 ± 29.2	39.5 ± 43.8	0.41
Hypertension	11 (78.6)	23 (38.3)	0.007
Diabetes	3 (21.4)	8 (13.3)	0.44
Body mass index, kg/m^2^	24.9 ± 3.8	23.6 ± 3.8	0.25
C‐reactive protein, mg/dL	0.17 ± 0.18	0.16 ± 0.20	0.86
Neutrophil‐to‐lymphocyte ratio	2.53 ± 1.03	2.18 ± 0.79	0.17

Abbreviations: HMGB1, high‐mobility group box‐1; RCC, renal cell cancer.

^a^ Manual workers included manufacturing, construction, mining, and transportation workers; the other occupation group included professional, managerial, clerical, sales, and service workers, and those who were not actively engaged in paid employment (e.g., homemakers, students, unemployed, and class unknown workers).

**TABLE 2:** Numbers of high‐grade tumors and positive expressions for cytosolic high‐mobility group box‐1 among specific manual workers

Characteristics	Number (%)		
	Manufacturing	Construction	Other manual occupations
RCC patients in Kanagawa Cancer Registry^a^			
High‐grade RCC	5 (18.5)	6 (37.5)	3 (16.7)
Low‐grade RCC	22 (81.5)	10 (62.5)	15 (83.3)
Patients with low‐grade clear cell carcinoma in Kanto Rosai Hospital^b^			
Positive cytosolic HMGB1 expression	5 (37.5)	3 (75.0)	2 (100)
Negative cytosolic HMGB1 expression	3 (62.5)	1 (25.0)	0 (0)

Abbreviations: HMGB1, high‐mobility group box‐1; RCC, renal cell cancer.

^a^ In Kanagawa Cancer Registry, manual workers included manufacturing (*n* = 27), construction (*n* = 16), and transportation workers (*n* = 18); the other occupation group included professional (*n* = 43), managerial (*n* = 25), clerical (*n* = 25), sales (n=35), and service workers (*n* = 15), and those who were not actively engaged in paid employment (e.g., homemakers, students, unemployed, and class unknown workers; *n* = 114).

^b^ In Kanto Rosai Hospital, manual workers included manufacturing (*n* = 8), construction (*n* = 4), mining (*n* = 1), and transportation workers (*n* = 1); the other occupation group included professional (*n* = 16), managerial (*n* = 9), clerical (*n* = 9), sales (*n* = 6), and service workers (*n* = 10), and those who were not actively engaged in paid employment (e.g., homemakers, students, unemployed, and class unknown workers; *n* = 10).

In a sensitivity analysis restricted to male patients, high‐grade tumors were more prevalent in manual workers compared with others in KCR: 24.1% (14/58) versus 11.5% (25/217, *p* = .01) with significantly elevated ORs (Figure 3). When excluding those in transportation, service and sales industries, the observed odds for high‐grade tumors among manual workers was significantly elevated, with some escalation of the effect size (Figure 3).

### 3.2 Positive cytosolic HMGB1 expression among low‐grade RCC in Kanto Rosai Hospital

For immunohistochemistry data, a positive cytosolic HMGB1 expression was more prevalent in manual workers compare with others (Table 1): 71.4% versus 38.3% (*p* = .03). The odds for positive cytosolic HMGB1 expression were elevated in manual workers (Table 3): the age and sex‐adjusted OR was 3.76 (95% CI, 1.03–13.7), which remained significant after additional adjustments for behavioral and chronic inflammatory factors.

**TABLE 3:** Occupational differences in positive expressions for cytosolic high‐mobility group box‐1 among patients with low‐grade clear cell carcinoma

Characteristics	Odds ratio (95% confidence interval)^a^		
	Crude	Age & sex adjusted	Additional adjustments
All, *n* = 74			
Manual worker^b^	4.02 (1.13–14.3)	3.76 (1.03–13.7)	8.56 (1.72–42.6)
Age, yrs	1.01 (0.97–1.05)	1.00 (0.96–1.04)	1.01 (0.97–1.06)
Female	0.61 (0.20–1.86)	0.75 (0.24–2.38)	0.70 (0.15–3.30)
Smoking, pack‐years	1.00 (0.97–1.02)		1.00 (0.97–1.04)
Drinking, drink‐years	1.00 (0.99–1.01)		1.00 (0.98–1.01)
Hypertension	0.77 (0.31–1.95)		0.40 (0.11–1.41)
Diabetes	0.67 (0.18–2.52)		0.52 (0.10– 2.77)
Body mass index, kg/m^2^	0.93 (0.82–1.05)		0.92 (0.79–1.07)
C‐reactive protein, mg/dL	2.05 (0.19–22.6)		3.31 (0.20–53.8)
Neutrophil‐to‐lymphocyte ratio	0.81 (0.46–1.42)		0.65 (0.34–1.25)
Men, *n* = 57			
Manual worker^c^	5.29 (1.27–22.0)	5.38 (1.25–23.2)	18.8 (2.57–137.0)
Age, yrs	1.01 (0.97–1.05)	1.00 (0.95–1.04)	1.01 (0.95–1.07)
Smoking, pack‐years	0.99 (0.97–1.02)		1.03 (0.99–1.07)
Drinking, drink‐years	1.00 (0.98–1.01)		1.00 (0.98–1.01)
Hypertension	1.23 (0.43–3.49)		0.53 (0.11–2.59)
Diabetes	0.15 (0.02–1.37)		0.03 (0.001–0.62)
Body mass index, kg/m^2^	0.96 (0.83–1.11)		0.95 (0.78–1.14)
C‐reactive protein, mg/dL	6.03 (0.17–218.5)		2.02 (0.02–250.1)
Neutrophil‐to‐lymphocyte ratio	0.74 (0.41–1.36)		0.48 (0.22–1.06)

^a^ Odds ratios and 95% confidence intervals for positive expression of cytosolic high‐mobility group box‐1 were estimated by logistic regression.

^b^ When excluding those in transportation, service and sales industries, the positive cytosolic HMGB1 expression was more prevalent in manual workers compared with others: 69.2% (9/13) versus 38.6% (17/44, *p* = .05), with the additionally‐adjusted OR of 6.35 (95% CI, 1.12–35.9).

^c^ The positive cytosolic HMGB1 expression was more prevalent in manual workers compared with others: 76.9% (10/13) versus 38.6% (17/44, *p* = .02).

In a sensitivity analysis restricted to male patients, the positive cytosolic HMGB1 expression was more prevalent in manual workers compared with others, with significantly elevated ORs (Table 3). Likewise, when excluding those in transportation, service and sales industries, the positive cytosolic HMGB1 expression was more prevalent in manual workers compared with others (Table 3).

Additionally, when restricted to the 62 specimens during 2012–2019, the occupational pattern of cytosolic HMGB1 expression was similar but not significant due to a small sample size: 66.7% (8/12) versus 40.0% (20/50, *p* = .096), with the maximally‐adjusted OR of 4.89 (95% CI, 0.91–26.3). Due to the data limitation, statistical assessment was not possible for the 12 specimens during 2008 to 2012 (the positive cytosolic HMGB1 expression was, respectively, observed in 2 out of 2 among manual workers and 3 out of 10 among others).

## 4 DISCUSSION

As far as we are aware, this study is the first to suggest occupational differences in tumor grades and immunohistochemistry for RCC, using data from a population‐based cancer registry and nested inpatients of a designated hospital simultaneously. Compared with RCC patients of other occupational groups, those of manual workers had double the risk for high‐grade tumors. In addition, even among low‐grade cancer patients, blue‐collar industries such as manufacturing and constructions tend to be associated with higher risks for histological malignancies confirmed by HMGB1, after adjusting for behavioral and chronic inflammatory factors. Therefore, our findings suggest lifestyle related factors and carcinogenesis on the tumor grade of RCC may differ by occupational background.

For occupational disparities in RCC incidence, specific occupational exposures to carcinogens of industrial agents (e.g., trichloroethylene and heavy metal) partly play a role.^6^, ^7^, ^8^, ^9^ However, on top of this traditional occupational disparity linked to specific carcinogens, occupations are known to play as a proxy for socioeconomic status for cancer incidence. For RCC incidence, established lifestyle‐related risks are smoking, hypertension, diabetes, and obesity.^1^, ^4^ Traditionally, workers in low socioeconomic occupations such as manual workers in blue‐collar industries are more likely to have these risks. In Japan, we recently reported that managers and professionals in service industries are more likely to report occupational stress and hypertension, and to have a higher risk for RCC *incidence* compared with blue‐collar workers.^2^ In recent studies from South Korea and Nordic countries, the similar pattern has been observed.^8^, ^9^ However, occupational impacts on tumor grades have remained inconclusive. In the present study, we found that high‐risk histological potentials were associated with manual workers. Therefore, different aspects of occupations may play a role in tumor grades.

The possible mechanisms underlying occupational differences in tumor grades in RCC remain unclear and scarce. Meanwhile knowledge on socioeconomic gaps in tumor grades has been accumulating in other urological cancers. For instance, occupational differences in exposure to hazardous chemicals might be possible. In a hospital‐based study with 454 patients with bladder cancer in the United Kingdom, high‐grade tumors were more common among blue‐collar workers, in particularly those working in manufacturing and transportation, compared with other occupational groups.^29^

Regarding HMGB1 functions, including inflammation, proliferation, and tissue regeneration in the pathogenesis of renal diseases, the main function of HMGB1 in cytoplasm is known to limit apoptosis.^13^ HMGB1 can translocate from nucleus to cytoplasm under certain stress, and is suggested to accelerate kidney damage and promote RCC development.^13^, ^14^, ^30^ Although mechanisms of occupation‐related tumor grades through HMGB1 have not been explained, it is possible that job stress in blue‐collar workers (e.g., lower job controls)^31^ might induce chronic inflammation in micro‐environment of renal cells and in turn increase the risk of high‐grade cancer potentials. Hence, future studies are expected to explore biological pathways of higher tumor grades from occupational stress in combination with lifestyle‐related risks to HMGB1 expression in RCC.

This study has several limitations. First, although pathological data from a population‐based cancer registry and a designated hospital were diagnosed by pathologists and updated to the latest version to be consistent with changes in coding practice, these were not based on a central pathology review. Therefore, information bias on tumor grades might be possible. On the other hand, misclassification of tumor grade is unlikely to be differential with respect to our exposure of interest (industry type), and hence, the likely direction of bias is toward the null. Second, our sample size was small, and the results of multivariable regression analysis adjusted for several behavioral and chronic inflammatory covariates should be interpreted cautiously. Also, we could not evaluate the association for non‐CCC subtypes. Additionally, immunohistochemistry data for normal renal tissues were not available due to the limitation of our study design. Stainability were changeable due to different storage duration in fixing solution and antibody. Data were also limited for occupation, with a large fraction (90%) missing, so that imputation was not possible. Third, KCR does not collected occupational information down to the 3‐digit code of the International Standard Classification of Occupations.^32^

Although the national cancer registry in Japan does not collect relevant socioeconomic indicators (i.e., occupation, educational attainment, or incomes), we found occupational differences in RCC tumor grades for the first time with a KCR dataset, which partly included individual‐level occupations. Additionally, in a recent study, cancer patients of manual workers had 1.25 times higher odds for advanced cancer stage,^28^ which also suggests the existence of socioeconomic disparities in health behaviors including delayed diagnosis with non‐cancer screening in Japan. Hence, further studies should address occupational backgrounds in histological characteristics, trying to use data linkage approaches.^18^, ^33^ In conclusion, occupational disparities in RCC tumor grades appeared to exist in Japan. Further integrated perspectives of clinical pathology, occupational medicine, and social epidemiology are warranted to elucidate the determinants of occupational differences in RCC carcinogenesis.

## DISCLOSURE

*Ethical approval*: The ethical committee of Dokkyo Medical University approved this study (Protocol Number 2020–004). *Informed consent*: N/A. *Registry and the Registration No*. *of the study*/*Trial*: N/A. *Animal Studies*: N/A. *Conflict of interest*: The authors declare no potential conflicts of interest.

## AUTHOR CONTRIBUTIONS

Conceptualization: Masayoshi Zaitsu, Takumi Takeuchi, Yasuki Kobayashi, and Ichiro Kawachi, Methodology: Masayoshi Zaitsu, Takumi Takeuchi, Masaaki Zaitsu, Akiko Tonooka, Toshimasa Uekusa, Yasuki Kobayashi, and Ichiro Kawachi, Formal Analysis: Masayoshi Zaitsu, Takumi Takeuchi, Akiko Tonooka, and Toshimasa Uekusa, Funding Acquisition: Masayoshi Zaitsu and Yasuki Kobayashi, Writing—Original Draft Preparation: Masayoshi Zaitsu, Writing—Review & Editing: Masayoshi Zaitsu, Takumi Takeuchi, Masaaki Zaitsu, Akiko Tonooka, Toshimasa Uekusa, Yudai Miyake, Yasuki Kobayashi, Gen Kobashi, and Ichiro Kawachi, Study supervision: Masayoshi Zaitsu.

## Data Availability

The data used in the analyses reported in this study are available from the corresponding author upon reasonable request. Requests to access the original database of Kanagawa Cancer Registry can be made by following the procedures described at the following URL (http://www.pref.kanagawa.jp/docs/nf5/ganntaisaku/know‐about‐gan/ganntouroku‐deta‐start.html).
